# Glomerulopathy in the KK.Cg-*A^y^*/J Mouse Reflects the Pathology of Diabetic Nephropathy

**DOI:** 10.1155/2013/498925

**Published:** 2013-04-24

**Authors:** Stephen P. O'Brien, Mandy Smith, Hong Ling, Lucy Phillips, William Weber, John Lydon, Colleen Maloney, Steven Ledbetter, Cynthia Arbeeny, Stefan Wawersik

**Affiliations:** Tissue Protection and Repair, Genzyme, A Sanofi Company, 49 New York Ave., Framingham, MA 01701, USA

## Abstract

The KK.Cg-*A* ^*y*^/J (KK-*A* ^*y*^) mouse strain is a previously described model of type 2 diabetes with renal impairment. In the present study, female KK-*A* ^*y*^ mice received an elevated fat content diet (24% of calories), and a cohort was uninephrectomized (Unx) to drive renal disease severity. Compared to KK-*a*/*a* controls, 26-week-old KK-*A* ^*y*^ mice had elevated HbA1c, insulin, leptin, triglycerides, and cholesterol, and Unx further elevated these markers of metabolic dysregulation. Unx KK-*A* ^*y*^ mice also exhibited elevated serum BUN and reduced glomerular filtration, indicating that reduction in renal mass leads to more severe impairment in renal function. Glomerular hypertrophy and hypercellularity, mesangial matrix expansion, podocyte effacement, and basement membrane thickening were present in both binephric and uninephrectomized cohorts. Glomerular size was increased in both groups, but podocyte density was reduced only in the Unx animals. Consistent with functional and histological evidence of increased injury, fibrotic (fibronectin 1, MMP9, and TGF**β**1) and inflammatory (IL-6, CD68) genes were markedly upregulated in Unx KK-*A* ^*y*^ mice, while podocyte markers (nephrin and podocin) were significantly decreased. These data suggest podocyte injury developing into glomerulopathy in KK-*A* ^*y*^ mice. The addition of uninephrectomy enhances renal injury in this model, resulting in a disease which more closely resembles human diabetic nephropathy.

## 1. Introduction

Diabetic nephropathy (DN) is the leading cause of end-stage renal disease (ESRD) and is associated with high cardiovascular risk and significant morbidity and mortality [1, 2]. Disease progression is difficult to predict, as rates of decline in glomerular filtration rate (GFR) are variable in this patient population and only one-third of diabetic patients, the majority of whom have type 2 diabetes (T2D), develop progressive renal failure. The pathogenesis of DN is mediated by a complex interplay of genetic and environmental modifiers resulting in hemodynamic and structural changes in the kidney that contribute to progressive functional loss in both the glomerulus and tubular-interstitial epithelium [3, 4]. Central to disease progression is glomerular injury, with pathological changes including glomerular hypertrophy, glomerular basement membrane (GBM) thickening, mesangial matrix expansion, and subsequent glomerulosclerosis [3]. Podocytes are critical to the functional glomerular filtration barrier and are particularly sensitive to damage by the “diabetic milieu” of dysglycemia, dyslipidemia, hemodynamic changes, and inflammation [5, 6]. In both T1 and T2D patients, podocyte detachment and loss are associated with decline in glomerular function [7–9]. 

While rodent DN models have provided significant insight into renal disease pathophysiology, no model captures all the features of human DN [10–13], creating an obstacle to understanding disease etiology and to developing effective treatments [14]. To address this, the NIH-sponsored Animal Models of Diabetic Complications Consortium (AMDCC) committee was formed to establish phenotyping standards and validation criteria for available murine models of DN (http://www.diacomp.org/). The committee defined criteria for mouse models of DN that reflect human disease, including greater than 50% decline in GFR over the lifetime of the animal model, 10-fold increase in albuminuria relative to age- and gender- matched controls, and renal pathology characterized by advanced mesangial matrix expansion, arteriolar hyalinosis, and glomerular basement membrane thickening [10, 11]. While numerous diabetic murine models were phenotyped and met some of the criteria, no one model fulfilled all of the AMDCC criteria. The committee consequently recommended the use of a “suite” of mouse models, each recapitulating individual features of renal disease in diabetic patients [11, 15].

 One of the mouse models not extensively examined by the AMDCC is the KK-*A* ^*y*^ strain. This model exhibits marked obesity, glucose intolerance, severe insulin resistance, dyslipidemia, and hypertension [16–18]. KK-*A* ^*y*^ mice also develop renal disease characterized by moderate albuminuria with mild glomerular pathology and podocyte loss [19–21]. Several therapeutic interventions have been reported to reduce albuminuria and improve renal pathology in this model, including renin-angiotensin blockage [18, 22, 23], statin therapy [24], and vitamin D [25]. However, direct measurement of GFR in this model has not been reported and is an important component, as decline in GFR is the benchmark of disease progression, and prevention of decline in GFR is a key efficacy endpoint in therapeutic clinical trials [26, 27].

The goal of this study was to increase renal disease severity in the KK-*A* ^*y*^ model by multiple “hits,” each reflecting a known factor contributing to diabetes and its complications in humans. We combined dietary manipulation and reduction in renal mass to increase renal injury in this model. Together, these environmental modifiers exacerbate glomerular and tubulointerstitial pathology, increase podocyte loss, and reduce GFR in KK-*A* ^*y*^ mice, and our findings support the use of this multifactorial approach in developing appropriate models for human diabetic nephropathy.

## 2. Materials and Methods

### 2.1. Experimental Animals

All animal studies followed the principles of laboratory animal care established by the National Institutes of Health and the Institutional Animal Care and Use Committee (IACUC). Female KK.Cg-*A* ^*y*^/J (further referred as KK-*A* ^*y*^) and littermate control KK-*a/a* mice were purchased from Jackson Laboratories (Bar Harbor, ME, USA) and acclimated for 3 weeks before the beginning of the study. Cohorts of mice were uninephrectomized at 8 weeks of age. At 9 weeks, animals were placed on semipurified control diet (D08112307) containing 12% k/cal fat or a moderately high-fat diet containing 24% k/cal fat and 0.2% cholesterol (D10011701) (Research Diets Inc., St. Louis, MO, USA). Mice were randomized according to albumin/creatinine at 12 weeks of age and were sacrificed at 26 weeks of age.

### 2.2. Physiological and Biochemical Characterization

Blood pressure was measured at 11 and 22 weeks of age by a noninvasive tail cuff CODA system (Kent Scientific, Torrington, CT, USA) after the mice were externally prewarmed for 10 min at 37°C. Animals were trained once daily, 4 days prior to recorded measurements. Eight to twenty recordings were taken for each measurement. 

Fasting blood samples were collected by retroorbital sinus bleeds. Collected samples were separated to serum or EDTA plasma or kept as whole blood. Glycated hemoglobin (HbA1c), serum and urine creatinine, blood urea nitrogen (BUN), serum cholesterol, and triglycerides were measured using a Cobas 400 plus bioanalyzer (Roche Diagnostics, IN, USA). Blood was also collected from the tail vein from unanesthetized, nonfasted animals for the measurement of blood glucose using a glucometer. Body weight (BW) was measured weekly. Urine samples were collected for 24 h using metabolic cages. Urine albumin was measured by immunoassay Albuwell M (Exocell Inc., Philadelphia, PA, USA). Immuno-ELISA according to manufacturer's instructions was used to measure serum adiponectin and insulin (ALPCO Diagnostics, Salem, NH, USA), PAI-1 (Molecular Innovations, Novi, MI, USA), urine nephrin (Exocell Inc., Philadelphia, PA, USA), and KIM-1 (Immunology Consultants Laboratory, Inc. Portland, OR, USA).

### 2.3. Glomerular Filtration Rate

Glomerular filtration rate (GFR) was performed at 23 weeks of age using a FIT-GFR Test Kit for Inulin according to the manufacturer's instructions (BioPal, Worcester, MA, USA). A 5 mg/kg bolus intraperitoneal injection of inulin was given, followed by serial saphenous bleeds at 30, 60, and 90 minutes. Serum was isolated and quantified on inulin ELISA. Inulin serum clearance was determined by nonlinear regression using a one-phase exponential decay formula (*y* = *Be* ^−*bx*^), and GFR was calculated (GFR = ((*I*)/(*B*/*b*))/KW, where *I* is the amount of inulin delivered by the bolus injection, *B* is *y*-intercept, *b* is the decay constant, *x* is time, and KW is kilo weight of the animal).

### 2.4. Histological Assessment

 Formalin-fixed, paraffin-embedded kidneys were sectioned at 3 microns and stained with hematoxylin and eosin (H&E), periodic acid-Schiff (PAS), and Masson's Trichrome for histological analysis. Slides were evaluated by a veterinary pathologist. Glomerular and tubular pathology, interstitial inflammation including presence of CD68 immunolabeled macrophages, and interstitial fibrosis were semiquantitatively scored on a scale of 0–4 as follows: 0 = normal; 1 = mild; 2 = moderate; 3 = marked; 4 = severe. Statistical analysis was performed using the nonparametric Kruskal-Wallis test followed by Dunn's multiple comparison test. 

### 2.5. Immunohistochemistry

Immunohistochemistry was performed on paraffin-embedded kidney sections. Anti-CD68 immunohistochemistry staining was performed on a Leica Bond MAX automated immunostainer (Leica Microsystems Inc. Buffalo Grove, IL, USA). Tissue sections were dewaxed, treated with Proteinase K enzyme, and followed by blocking with peroxidase and then serum. Slides were incubated in CD68 Clone Fa-11 (Abcam, Cambridge, MA, USA) antibody for 30 minutes, followed by rabbit anti-rat antibody, then a goat anti-rabbit HRP polymer for 15 minutes. Chromogen visualization was performed using 3,3′-diaminobenzidine tetrahydrochloride (DAB) for 3–5 minutes, followed by hematoxylin counterstain (all components were from Leica, unless otherwise noted). 0.05% Tween 20/Tris-buffered saline (DAKO, Carpinteria, CA, USA) washes were performed between all steps. Podocyte counting was assessed using anti-WT1 (Wilms Tumor 1) clone 6F-H2 at 1 : 100 dilution (Dako). Immunohistochemistry was performed on a Leica Bond MAX automated immunostainer (Leica Microsystems Inc. Bannockburn, IL, USA). 0.05% Tween 20/Tris-buffered saline (DAKO) washes were performed between all steps. Tissue sections were dewaxed and treated with Proteinase K enzyme then peroxidase. Tissues were then treated with rodent block (BioGenex, Fremont, CA, USA), anti-WT-1 primary antibody was detected using mouse on mouse HRP polymer. Chromogen visualization was performed using 3,3′-diaminobenzidine tetrahydrochloride (DAB) for 5 minutes, followed by hematoxylin counterstain and dehydration through increasing ethanol-water gradient to xylene and mounted in Permount (Fisher Scientific, Pittsburg, PA, USA). Whole kidney sections were imaged using Aperio ScanScope (Aperio Technologies, Vista, CA, USA). Greater than 50 glomeruli per kidney section were quantitated for the number of WT-1-positive (brown) and WT-1-negative cells (blue). Software analysis was done using custom algorithm on Spectrum Version 11.0.0.725 (Aperio Technologies).

### 2.6. Transmission Electron Microscopy

For ultrastructural analysis, 1 mm^3^ samples of kidney cortex were fixed in 3% glutaraldehyde in 0.2 M cacodylate buffer (Electron Microscopy Sciences, Hatfield, PA, USA) and embedded in epon. Semithin 1 micron sections were stained with Richardson's stain (made up of methylene blue CI-52015, azure II, and borax, (Sigma, St. Louis, MO, USA)) and evaluated via high resolution light microscopy. Ultrathin sections were cut at 70 nm and mounted on 200 mesh copper grids. The grids were stained on a Leica EM AC20 Stainer (Leica Microsystems) with 0.5% aqueous uranyl acetate and 3% lead citrate solution (Leica). Images were acquired on a JEOL JEM-1400 transmission electron microscope (JEOL; Peabody, MA, USA) using a Gatan 785 Erlangshan ESW1000 digital camera (Gatan, Inc.; Pleasanton, CA, USA).

### 2.7. Transcriptional Analysis

Quantitative transcriptional analysis of kidney tissue from animals at 26 weeks of age was performed on TaqMan Custom Array microarrays using 7900HT Fast real-time PCR system. Kidney mRNA purification was performed by homogenizing 50–100 mg of tissue in 1 mL of TRIzol reagent. Samples were left for 5 minutes at 15 to 30°C followed by the addition of 0.2 mL chloroform (Sigma). Samples were mixed vigorously, left at room temperature for 3 minutes followed by centrifugation at 12,000 ×g for 15 minutes at 4°C. The aqueous phase was taken, and RNA was precipitated with 0.5 mL of isopropyl alcohol (Sigma). The pellet was washed once with 1 mL 75% ethanol, air-dried, and redissolved in RNase-free water. Samples were DNAse treated using TurboDNase according to manufacturer's instructions. RNA was qualified on an Agilent 2100 Bioanalyzer (Agilent Technologies, Santa Clara, CA, USA). All items were obtained from Life Technologies, Grand Island, NY, USA unless otherwise noted.

## 3. Results

### 3.1. Uninephrectomized KK-A^y^ Mice Are Obese and Exhibit Evidence of Metabolic Abnormalities

Diabetes and renal injury are more severe in male versus female KK-*A* ^*y*^ mice [28] and, as a consequence, preclinical studies typically focus on males. However, male KK-*A* ^*y*^ develop obstructive uropathy and hydronephrosis [29] and exhibit impaired glucose tolerance [28]. To avoid these urological issues, all studies described here use female mice. This also allowed us to use KK-*a/a* females as nondiabetic, strain-matched controls, as female KK-*a/a* mice maintain normal glycemic control, while male KK-*a/a* are insulin resistant [30]. 

**Figure 1:**
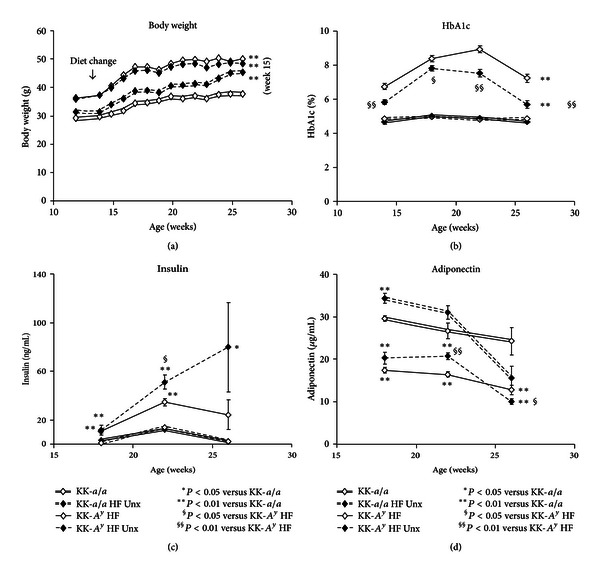
Obesity, glucose intolerance, insulin resistance, and hypoadiponectinemia in KK-*A* ^*y*^ mice. (a) Prior to diet change of a moderately high-fat diet at 12 weeks, KK-*A* ^*y*^ were significantly heavier than KK-*a/a* control animals. After 3 weeks on modified diet, KK-*a/a* control animals became obese, but never reached the same level as KK-*A* ^*y*^ (*P* < 0.01). (b) Throughout the study, glycated hemoglobin was elevated in KK-*A* ^*y*^ mice (compared to KK-*a/a *controls, *P* < 0.01). KK-*a/a* mice maintain normal long-term glucose homeostasis. Hyperglycemic uninephrectomized KK-*A* ^*y*^ (KK-*A* ^*y*^ + Unx) animals had lower HbA1c than birenal KK-*A* ^*y*^ (*P* < 0.05). (c) All KK-*A* ^*y*^ had elevated insulin, but greater hyperinsulinemia was evident in the KK-*A* ^*y*^ + Unx group, where insulin increased progressively throughout the study. (d) Consistent with obesity, all KK-*A* ^*y*^ mice had lower adiponectin levels at 16 weeks of age, which were further decreased by 26 weeks. The observed decline in adiponectin levels in KK-*a/a* Unx and high-fat diet between 22–26 weeks was not statistically different from the KK-*a/a* control. The KK-*A* ^*y*^ + Unx adiponectin levels were significantly lower than KK-*A* ^*y*^ binephric mice.

While on standard chow, KK-*A* ^*y*^ mice were significantly heavier than control or uninephrectomized (Unx) KK-*a/a *animals (Figure 1(a)). After switching to the elevated fat diet, KK-*a/a* + Unx mice gained weight relative to KK-*a/a *controls, but were less obese than both KK-A^*y*^ groups. Blood pressure, measured in 11-and 22-week-old mice, was unchanged in both the KK-*a/a *and KK-*A* ^*y*^ + Unx groups, and no difference was observed between the two cohorts. At 11 weeks, KK-*a/a* mean arterial pressure (MAP) was 85.6 ± 3.7 mmHg, while KK-*A* ^*y*^ + Unx MAP was 83.5 ± 2.2 mmHg. At 22 weeks, MAP in KK-*a/a* mice was 84.0 ± 3.3 mmHg and KK-*A* ^*y*^ + Unx MAP was 86.5 ± 3.5 mmHg. 

In KK-*a/a *controls, glycated hemoglobin (HbA1c) was unaffected by the switch to an elevated fat diet (Figure 1(b)). Serum HbA1c levels were elevated in all KK-*A* ^*y*^ mice on standard chow, and serum HbA1c further increased after the switch to the elevated fat diet, with peak levels occurring between 5 and 10 weeks after diet change. While serum HbA1c levels were higher in uninephrectomized KK-*A* ^*y*^ (KK-*A* ^*y*^ + Unx) mice than in KK-*a/a*, these levels were consistently lower compared to birenal KK-*A* ^*y*^, a likely consequence of elevated insulin levels in the KK-*A* ^*y*^ + Unx animals (Figure 1(c)). Consistent with the HbA1c levels, blood glucose in nonfasted, unanesthetized animals was also significantly elevated in the KK-*A* ^*y*^ + Unx group relative to KK-*a/a* mice (318.8+/26.3 versus 222.3+/−20.3, *P* < 0.01). However, to avoid undue stress caused by unanesthetized blood collection, and because we note short-term variability in glucose values as mice are moved into the metabolic cages for urine collection, glycemia was followed in this study using HbA1c. Serum adiponectin levels were reduced at 16 weeks of age in both KK-*A* ^*y*^ groups, which were further decreased at 26 weeks (Figure 1(d)). Interestingly, uninephrectomy in the KK-*a/a* mice was also associated with a sharp decline in adiponectin levels between 20 and 26 weeks of age.

**Figure 2:**
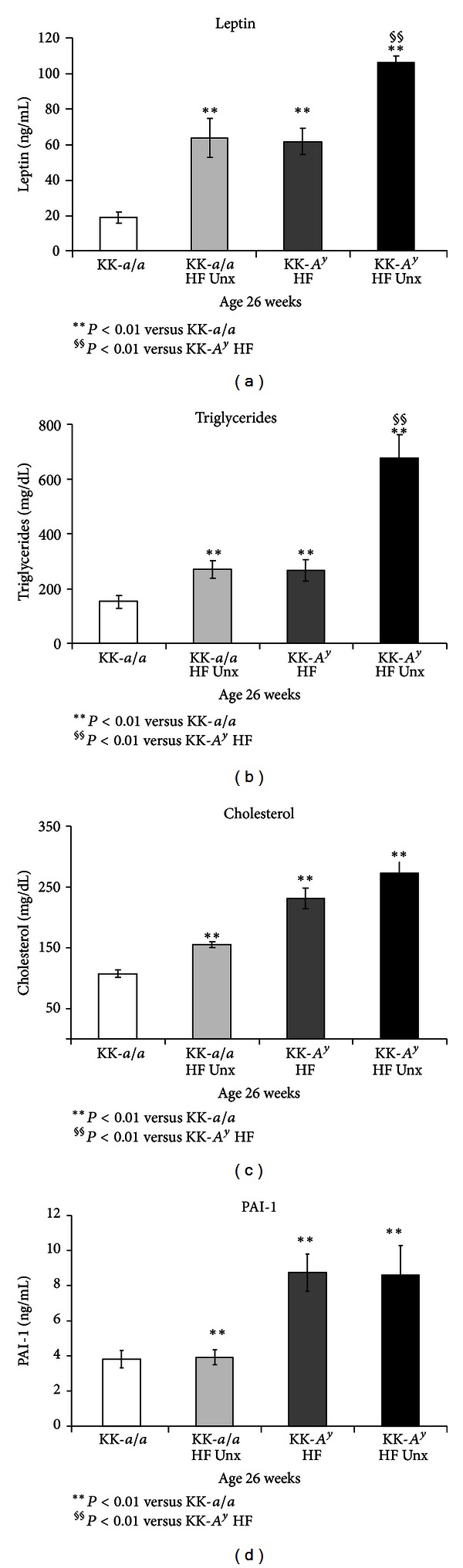
Altered serum cytokines and serum lipids in KK-*A* ^*y*^ mice. (a) Leptin levels were elevated in KK-*A* ^*y*^ (versus KK*-a/a* control). Addition of uninephrectomy (Unx) elevated serum leptin, both in KK*-a/a* and in KK-*A* ^*y*^ mice (*P* < 0.01). (b) A similar profile was observed for serum triglycerides, which were elevated in KK-*A* ^*y*^ (versus KK-*a/a* control). As with leptin, Unx further elevated triglycerides both in KK*-a/a* and in KK-*A* ^*y*^ mice (*P* < 0.01). (c) Relative to KK-*a/a *controls, KK-*a/a* + Unx, KK-*A* ^*y*^, and KK-*A* ^*y*^ + Unx mice all showed markedly elevated serum cholesterol (*P* < 0.001). (d) Serum PAI-1 levels were elevated in KK-*A* ^*y*^ mice compared to age-matched KK-*a/a* animals (*P* < 0.01). However, PAI-1 levels in KK-*A* ^*y*^ were unaffected by Unx.

 Consistent with increased body weight, serum lipids and adiposity-associated cytokines were elevated in KK-*A* ^*y*^ mice. Serum leptin was elevated 2-fold relative to KK-*a/a* controls in binephric KK-*A* ^*y*^ mice and approximately 5-fold in KK-*A* ^*y*^ + Unx animals (Figure 2(a)). Serum leptin was also elevated by Unx in both KK*-a/a* and KK-*A* ^*y*^. Serum triglyceride levels show a similar profile: both KK-*a/a* and KK-*A* ^*y*^ uninephrectomized mice were hypertriglyceridemic compared to binephric animals, and triglycerides were higher in KK-*A* ^*y*^ mice than in comparable KK-*a/a*. KK-*A* ^*y*^ + Unx triglycerides were elevated 4.5-fold versus binephric KK-*a/a* controls (Figure 2(b)). Serum cholesterol levels were also markedly elevated in KK-*A* ^*y*^ + Unx mice (Figure 2(c)) and further increased by uninephrectomy, an effect consistent with that observed for serum leptin and triglycerides. In contrast, serum PAI-1 levels were not affected by uninephrectomy (Figure 2(d)), but were significantly elevated in KK-*A* ^*y*^ mice relative to KK-*a/a. *Taken together, these data are consistent with the metabolic profile of human T2DN [2, 31, 32].

### 3.2. Urine and Serum Biomarkers Indicate Renal Injury in KK-A^y^ and KK-A^y^ + Unx Mice

**Figure 3:**
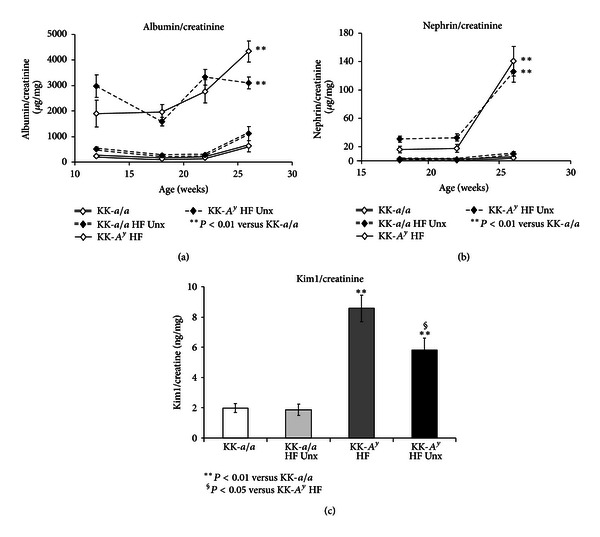
Urine markers indicate glomerular injury in KK-*A* ^*y*^. (a) KK-*A* ^*y*^ mice exhibited consistent and progressive albuminuria, measured as albumin/creatinine ratio (ACR) (*P* < 0.01 at all timepoints versus KK-*a/a*). No difference was observed in ACR between KK-*A* ^*y*^ and KK-*A* ^*y*^ + Unx. (b) Glomerular injury in KK-*A* ^*y*^ mice was suggested by progressive elevation of the podocyte-specific protein nephrin in the urine. Again, urine nephrin levels were comparable in KK-*A* ^*y*^ and KK-*A* ^*y*^ + Unx mice (*P* < 0.01 versus KK-*a/a*). (c) When compared to KK-*a/a*, the renal tubular marker Kim1 was also elevated in KK-*A* ^*y*^ mice. Interestingly, Kim1 levels in KK-*A* ^*y*^ + Unx animals were lower than in KK-*A* ^*y*^ mice.

KK-*A* ^*y*^ mice exhibit consistent and progressive albuminuria, measured as albumin/creatinine ratio (ACR) (Figure 3(a)). ACRs were comparable in both KK-*A* ^*y*^ and KK-*A* ^*y*^ + Unx, suggesting that uninephrectomy does not affect albuminuria in KK-*A* ^*y*^ animals. Urinary excretion of the podocyte protein nephrin, recently identified as a biomarker of glomerular injury in both humans and mice [33, 34], was elevated in both KK-*A* ^*y*^ and KK-*A* ^*y*^ + Unx mice at 26 weeks (14 weeks after the switch to elevated fat diet), with a trend towards nephrinuria first detected at 22 weeks (Figure 3(b)). As with ACR, we observed no difference in urinary nephrin/creatinine ratio in intact and uninephrectomized mice, suggesting glomerular injury in both KK-*A* ^*y*^ groups.

We also examined the concentration of KIM-1, a marker of ongoing renal tubular injury [35, 36], in urine collected from 26-week-old mice (Figure 3(c)). Relative to KK-*a/a *animals, KIM-1 was elevated in both KK-*A* ^*y*^ and KK-*A* ^*y*^ + Unx mice. Interestingly, KIM-1 levels in KK-*A* ^*y*^ + Unx animals were lower than in their binephric counterparts, possibly as a consequence of the reduced renal mass in uninephrectomized mice. 

**Figure 4:**
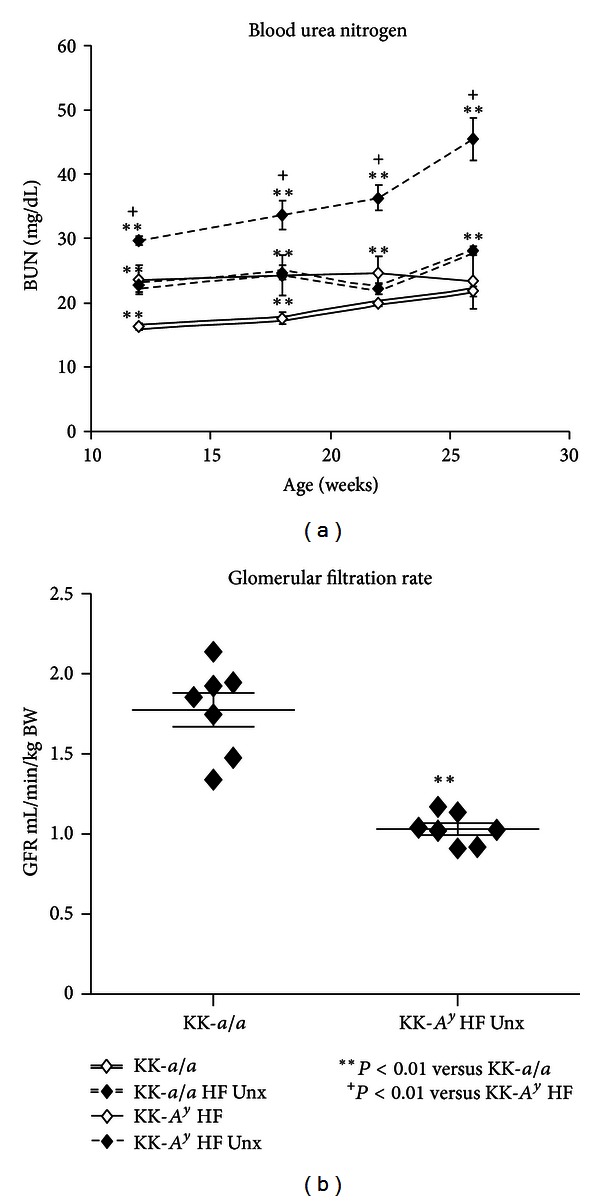
Decreased renal function in KK-*A* ^*y*^ is exacerbated by uninephrectomy. (a) Compared to KK-*a/a* mice, blood urea nitrogen (BUN) is slightly elevated in KK-*A* ^*y*^ and KK-*a/a* + Unx, though this difference is not statistically significant at later time points in the KK-*a/a* + Unx group. Uninephrectomy further elevates BUN in KK-*A* ^*y*^, with BUN increased by twofold, indicating mild uremia in KK-*A* ^*y*^ + Unx animals. (b) Renal function, as measured by glomerular filtration rate (GFR), is reduced by 32% in KK-*A* ^*y*^ + Unx mice compared to KK-*a/a* littermate controls (*P* < 0.001).

Elevated ACR and urine nephrin and KIM-1 concentrations suggest renal glomerular and tubular injury in KK-*A* ^*y*^ mice. To determine whether renal function was affected, we examined serum blood urea nitrogen (BUN) levels in these animals (Figure 4(a)). BUN was of normal range in KK-*a/a *mice, but was slightly elevated in KK-*a/a* + Unx and KK-*A* ^*y*^ animals. Mice in the KK-*A* ^*y*^ + Unx group, however, showed substantially greater increases in BUN, reaching an average of 45.3 mg/dL at 26 weeks (*P* < 0.0001 versus both KK-*A* ^*y*^ and KK-*a/a*). These BUN values suggest impaired renal function in KK-*A* ^*y*^ + Unx mice, and we therefore directly measured glomerular filtration rate (GFR) in this group. We found a 32% reduction in GFR in KK-*A* ^*y*^ + Unx mice compared to KK-*a/a* littermate controls, a reduction roughly equivalent to late stage 2 CKD in human patients. Thus, despite the finding that urine biomarkers revealed little difference in the extent of renal damage between KK-*A* ^*y*^ and KK-*A* ^*y*^ + Unx mice, changes in BUN and GFR, both direct indicators of renal function, indicated substantial renal impairment only in KK-*A* ^*y*^ + Unx mice.

### 3.3. Uninephrectomy Exacerbates Glomerulopathy and Renal Fibrosis Markers in KK-A^y^ Mice

**Figure 5:**
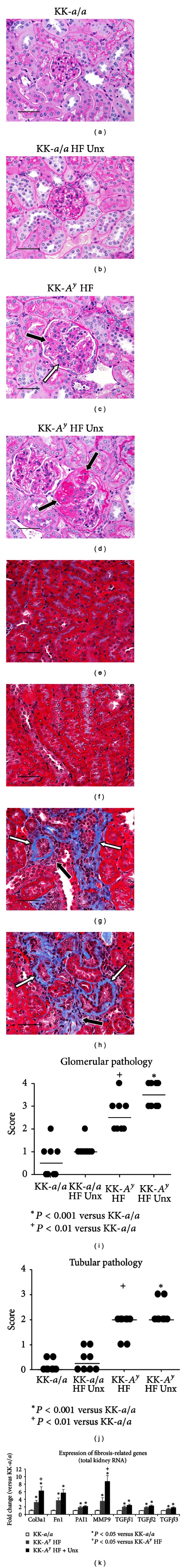
Glomerular injury in KK-*A* ^*y*^ diabetic mice. PAS staining in KK-*a/a *(a) and KK-*a/a* + Unx (b) mice revealed normal glomeruli. KK-*A* ^*y*^ mice (c) exhibited glomerular hypercellularity (white arrow) and mesangial matrix expansion (black arrow). KK-*A* ^*y*^ + Unx (d) had more severe glomerular mesangial matrix expansion (black arrow). Trichrome staining revealed normal tubules and interstitium in KK-*a/a *(e) and KK-*a/a* + Unx (f), whereas KK-*A* ^*y*^ (g) and KK-*A* ^*y*^ + Unx (h) had multifocal tubular atrophy with thickened basement membranes (white arrows) and mild interstitial fibrosis (black arrows). Scale bars represent 50 *μ*m. (i) In the semiquantitative pathological scoring, horizontal bars indicate group median score. The binephric KK-*A* ^*y*^ diabetic mice had a significantly greater group median score for glomerular pathology (*P* < 0.01 versus KK-*a/a* controls), with a trend toward exacerbation of pathology by uninephrectomy in the KK-*A* ^*y*^ + Unx group (*P* < 0.001 versus KK-*a/a* controls). (j) Both KK-*A* ^*y*^ groups had a significant increase in tubular pathology relative to KK-*a/a* controls (*P* < 0.01  KK-*A* ^*y*^; *P* < 0.001  KK-*A* ^*y*^ + Unx). (k) Whole kidney qRT-PCR indicated increased expression of fibrosis related genes by uninephrectomy in KK-*A* ^*y*^ versus KK-*a/a*. Expression of mRNA encoding Collagen 3a1 (Col3a1), fibronectin (FN1), serpine-1 (PAI-1), *matrix* metallopeptidase *9 (MMP9*), and the transforming growth factor beta-proteins (TGF*β*) 1–3 was significantly elevated in KK-*A* ^*y*^  versus KK-a/a. Addition of Unx to KK-*A* ^*y*^significantly elevated expression of Col3a and MMP9 versus KK-*A* ^*y*^(*P* < 0.01).

On histologic assessment, nondiabetic KK-*a/a* control mice exhibited minimal to mild glomerulopathy that was unchanged by elevated fat diet and Unx (Figures 5(a) and 5(b)). In contrast, diabetic KK-*A* ^*y*^ mice fed elevated fat diet exhibited a moderate to marked glomerulopathy (Figure 5(c)), characterized predominantly by segmental to global expansion of the mesangial matrix by PAS positive material and hypercellularity of the glomerular tuft. Semiquantitative scoring (Figure 5(i)) demonstrated a significant increase in the glomerulopathy in binephric diabetic KK-*A* ^*y*^ mice versus KK-*a/a* controls (*P* < 0.01), and this was further exacerbated by uninephrectomy in the KK-*A* ^*y*^ + Unx group (*P* < 0.001 vs. KK-*a/a* controls). Tubular lesions were rare to nonexistent in nondiabetic KK-*a/a* groups (Figures 5(e) and 5(f)). However, uninephric and binephric KK-*A* ^*y*^ groups exhibited multifocal tubular atrophy with thickened tubular basement membranes (Figures 5(g) and 5(h)), tubular dilation with or without luminal protein casts, and tubular mineralization at the corticomedullary junction. Overall, the tubular pathology was significantly greater in binephric KK-*A* ^*y*^ (*P* < 0.01) and uninephric KK-*A* ^*y*^ mice (*P* < 0.001, Figure 5(j)) relative to the KK-*a/a* controls. In the interstitium, KK-*A* ^*y*^ diabetic mice in both groups had multifocal infiltration of mixed inflammatory cells and mild fibrosis which typically surrounded severely affected glomeruli and/or tubules (Figures 5(g) and 5(h)). Inflammatory infiltrates were composed of macrophages, lymphocytes, and plasma cells admixed with occasional neutrophils. Although group median scores for interstitial fibrosis were similar in binephric and uninephric KK-*A* ^*y*^ groups, only the uninephric group showed a statistically significant increase relative to the KK-*a/a* controls (*P* < 0.001, not shown). 

 To further examine whether uninephrectomy affects fibrosis in KK-*A* ^*y*^ mice, we measured transcription of fibrosis-related genes in total kidney mRNA (Figure 5(k)). In KK-*A* ^*y*^ animals, we observed the upregulation of RNA encoding *Col3a1, Fn1, PAI-1, Mmp9, *and RNA encoding the TGF*β*1-3 ligands relative to KK-*a/a* controls. In KK-*A* ^*y*^ + Unx mice, we saw an additional statistically significant increase in *Col3a1* and *Mmp9* expression relative to binephric KK-*A* ^*y*^ animals, as well as a trend towards increased expression of the remaining fibrotic genes assessed. Taken together, these data indicate renal fibrosis in KK-*A* ^*y*^ mice and that uninephrectomy further exacerbates renal damage in this model.

### 3.4. Podocyte Injury in KK-A^y^ + Unx Mice

**Figure 6:**
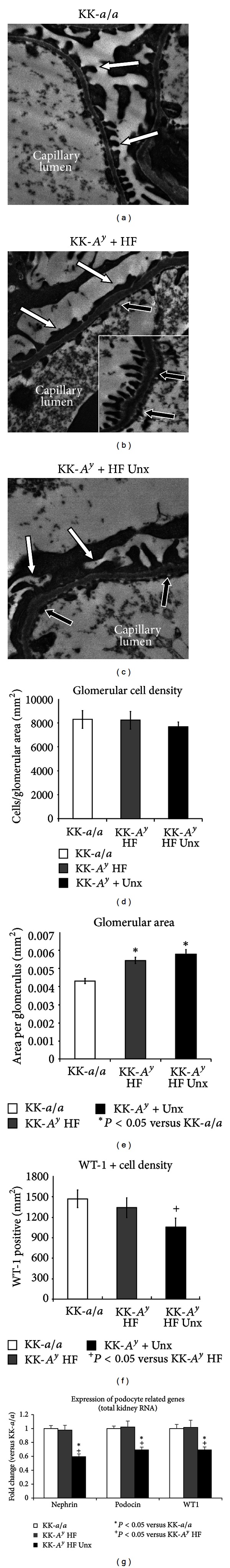
Podocyte injury in KK-*A* ^*y*^ + Unx mice. (a) At the ultrastructural level, KK*-a/a* controls had normal podocyte foot processes (white arrows) and a smooth, trilaminar glomerular basement membrane. In KK-*A* ^*y*^ (b) and KK-*A* ^*y*^ + Unx (c) mice, there was podocyte foot process effacement (white arrows) and areas of mild irregular GBM thickening (black arrows). Regions of uneffaced foot processes ((b), inset) were present in both KK-*A* ^*y*^ cohorts, and uninephrectomy did not induce discernable differences in the severity of glomerular basement membrane thickening. Podocyte counting was carried out using WT-1 as a specific podocyte marker. The total number of cells per glomerular area (d) was unchanged between any of the groups, but glomerular area was significantly increased in all KK-*A* ^*y*^ animals (e). In uninephrectomized KK-*A* ^*y*^ mice, the number of WT-1-positive cells per glomerular area was reduced by 28% (*P* < 0.05). The number of podocytes per glomerulus (independent of area) was also decreased in KK-*A* ^*y*^ (18% of total cells in KK*-a/a*, 16% in KK-*A* ^*y*^ HF, and 13% in KK-*A* ^*y*^ HF Unx (data not shown)) suggesting that loss of podocytes is not simply due to increased glomerular area. (g) qRT-PCR analysis of podocyte-specific genes further supports podocyte loss, revealing statistically significant decreases in nephrin, podocin, and WT-1 RNA expression in KK-*A* ^*y*^ HF Unx kidneys.

At the ultrastructural level, KK-*a/a *mice showed normal podocyte foot processes (Figure 6(a)) and a smooth, trilaminar glomerular basement membrane (GBM). Both KK-*A* ^*y*^ and KK-*A* ^*y*^ + Unx animals exhibited multifocal podocyte foot process effacement and areas of irregular thickening in the lamina densa of the GBM (Figures 6(b) and 6(c)). Because podocyte foot process effacement is reversible [37, 38], we used morphometric analysis of sections immunostained for the podocyte marker WT-1 to assess podocyte loss in KK-*A* ^*y*^ mice. No change was observed in average cell density per glomerulus in any of the groups examined (Figure 6(d)). However, we noted increased glomerular area in KK-*A* ^*y*^ and KK-*A* ^*y*^ + Unx mice. Furthermore, we observed a 28% decrease in the density of WT-1 positive cells per mm^2^ glomerular area in uninephrectomized KK-*A* ^*y*^ animals compared to KK-*a/a* mice (Figure 6(f)). This decrease in podocyte density was not observed in binephric KK-*A* ^*y*^ animals, a distinction consistent with the higher glomerular pathology scores in KK-*A* ^*y*^ + Unx mice (Figure 5(i)) and suggesting that addition of uninephrectomy exacerbates glomerular injury in the KK-*A* ^*y*^ model of diabetic nephropathy. 

 Examination of podocyte-specific transcripts further supports the conclusion that podocyte loss is observed only in KK-*A* ^*y*^ + Unx mice. Using quantitative RT-PCR, we measured expression of the podocyte-specific genes nephrin (*Nphs1), *podocin *(Nphs2)*, and WT-1 in total kidney mRNA (Figure 6(g)). We observed no difference in the expression of any of these genes between KK-*a/a* and binephric KK-*A* ^*y*^ mice, but note a statistically significant decrease in all three podocyte markers in KK-*A* ^*y*^ + Unx animals. These data are consistent with the decrease in podocyte density in KK-*A* ^*y*^ + Unx mice and suggest that uninephrectomy is necessary to induce podocyte loss in this model.

### 3.5. Increased Macrophage Infiltration and Inflammation in Uninephrectomized KK-A^y^ Mice

**Figure 7:**
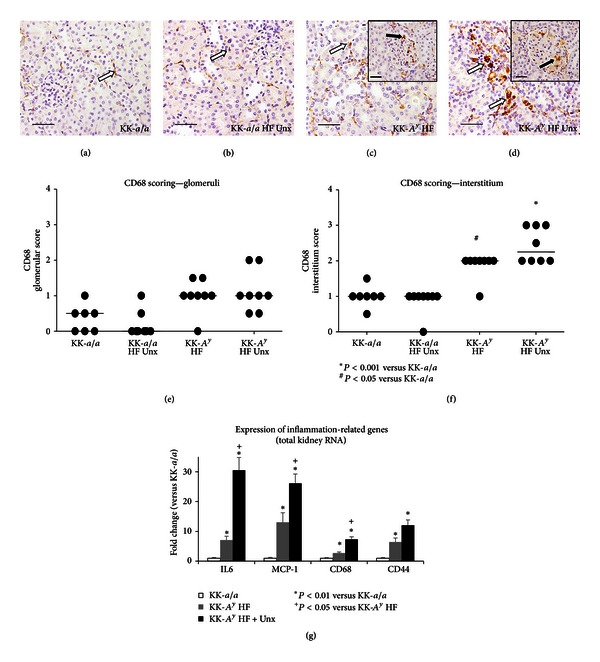
Increased inflammation in KK-*A* ^*y*^ mice.Macrophage infiltration was evaluated by CD68 immunohistochemistry. KK-*a/a* (a) and KK-*a/a *+ Unx (b) mice had scattered CD68^+^macrophages in the interstitium (white arrows) and rare CD68^+^cells in the glomeruli. KK-*A* ^*y*^ (c) and KK-*A* ^*y*^ + Unx (d) mice showed increased CD68^+^macrophage infiltration in the interstitium (white arrows) and occasional CD68^+^cells in glomeruli (inset, black arrows). Scale bars represent 50 *μ*m. Semiquantitative scoring of CD68^+^ cells revealed a modest increase in positively stained cells within glomeruli of KK-*A* ^*y*^ relative to the KK-*a/a* controls (e), additionally a statistically significant increase in interstitial CD68^+^ macrophages in both groups, with a slight enhancement in the KK-*A* ^*y*^ + Unx group (f). Horizontal bars indicate group median score and asterisk represents *P* value <0.001. (g) Gene expression was assayed by qRT-PCR using total kidney RNA from 26 week old mice KK-*A* ^*y*^, uninephrectomized KK-*A* ^*y*^, and control KK-*a/a* animals. qRT-PCR revealed increased expression of IL-6, MCP-1, CD68, and CD44 were significantly upregulated in KK-*A* ^*y*^ versus KK-*a/a* (*P* < 0.01). Uninephrectomy further exacerbated IL-6, MCP-1, and CD68 mRNA upregulation versus KK-*A* ^*y*^ (*P* < 0.05).

Macrophage infiltration and inflammation are increasingly recognized as important elements in diabetic glomerular disease [39]. To examine whether renal macrophage infiltration is present in the KK-*A* ^*y*^ model, immunohistochemistry for the macrophage marker CD68 was performed on kidney sections (Figure 7). In KK-*a/a *or KK-*a/a *+ Unx mice, there were scattered CD68^+^macrophages in the interstitium (Figures 7(a) and 7(b)) and rare CD68 positive cells in glomeruli. In contrast, binephric KK-*A* ^*y*^ mice (Figure 7(c)) exhibited a significant increase in interstitial CD68^+^ macrophages (*P* < 0.05 versus KK*-a/a* controls) and this was enhanced in the KK-*A* ^*y*^ + Unx group (*P* < 0001 versus KK*-a/a* controls, Figure 7(f)). Both KK-*A* ^*y*^ diabetic groups had low numbers of CD68^+^cells within glomeruli (Figure 7(e)). These may represent infiltrating macrophages or, alternatively, mesangial cells transforming to a macrophage phenotype. 

Immunohistochemical findings were corroborated by quantitative RT-PCR from total kidney mRNA which indicated a significant increase in the expression of CD68 and inflammation-associated markers IL-6, MCP-1, and CD44 in both KK-*A* ^*y*^ and KK-*A* ^*y*^ + Unx animals (Figure 7(g)). Expression of each of these markers was further increased in KK-*A* ^*y*^ + Unx mice, pointing to increased renal inflammation in this group. 

## 4. Discussion

The KK-*A* ^*y*^ mouse strain is glucose intolerant, severely insulin resistant, dyslipidemic, and hypertensive; all are characteristics of the “metabolic syndrome” phenotype of T2D patients [24–26]. Evidence of kidney injury is also present, including albuminuria, mesangial matrix accumulation, and GBM thickening [20]. However, as with many mouse models of diabetic nephropathy [10, 11], renal damage in KK-*A* ^*y*^ mice is mild and does not adequately capture key aspects of the human disease. To drive disease severity in KK-*A* ^*y*^, we provided a diet containing 24% of calories from fat and examined the effects of uninephrectomy (Unx) on metabolic and renal endpoints in this model. We found that while urinary markers of renal injury (albuminuria, nephrinuria, and urine Kim-1) were similar in KK-*A* ^*y*^ and KK-*A* ^*y*^ + Unx mice, uninephrectomized animals exhibited increased glomerular pathology, podocyte damage and loss, and renal inflammation. Furthermore, KK-*A* ^*y*^ + Unx mice had elevated serum BUN and reduced GFR, indicating the impairment of renal function. Our findings indicate that KK-*A* ^*y*^ + Unx mice have a more severe phenotype than previously reported in other DN models and, therefore, is a representative model of progressive glomerular injury and podocyte loss. 

Our strategy was to use reduction in renal mass and dietary manipulation in a strain which is genetically susceptible to renal disease to further exacerbate renal injury. First, KK-*A* ^*y*^ mice were uninephrectomized to induce glomerular hypertrophy, distortion of capillary architecture, and increased mechanical stress on the podocyte, factors which have been shown to exacerbate glomerulosclerosis [40, 41]. KK-*A* ^*y*^ mice were then fed a semipurified diet that is more representative of a normal human diet, with 24% of calories derived from fat. Excess dietary fat promotes tissue injury [42], and the very low fat content in normal rodent chow may consequently be protective. This elevated fat diet is also low in phytoestrogens, which are present in high levels in normal rodent chow [43] and can induce hormonal and antioxidant effects [44] that may blunt progressive injury. 

A significant advantage to using the KK-*A* ^*y*^ strain is that diabetes is polygenic and not due to a defect in leptin receptor signaling. This is analogous to human disease and in contrast to other obese diabetic models such as *db/db* and *ob/ob* mice [10, 17]. We found that both binephric and uninephrectomized KK-*A* ^*y*^ animals have elevated HbA1c, insulin, and leptin levels, suggesting both insulin and leptin resistance. We also observed a reduction in serum levels of the anti-inflammatory cytokine adiponectin [31, 45] and elevation of the proinflammatory cytokine PAI-1 [32, 46] in both cohorts of KK-*A* ^*y*^ mice, suggesting the presence of a proinflammatory state similar to human diabetic patients [47]. Inflammation may be exacerbated in KK-*A* ^*y*^ + Unx mice, as serum insulin and leptin levels (and, consequently, triglycerides) were further elevated in these animals. Taken together, these data indicate metabolic dysfunction and inflammation in both KK-*A* ^*y*^ and KK-*A* ^*y*^ + Unx cohorts, and suggest that uninephrectomy heightens inflammation in this model.

Urinalysis of KK-*A* ^*y*^ and KK-*A* ^*y*^ + Unx mice indicated renal injury in both groups. Albumin/creatinine ratios (ACRs) were elevated relative to KK-*a/a* controls, and increased levels of urine nephrin, a marker of podocyte injury in preclinical models and in human patients [48, 49], were also detected. Together, these findings suggest the impairment of the glomerular filtration barrier in KK-*A* ^*y*^ mice. Kim-1, a marker of proximal tubule injury [36], was also elevated in KK-*A* ^*y*^and KK-*A* ^*y*^ + Unx. We note, however, that Kim-1 in urine from KK-*A* ^*y*^ + Unx mice was slightly, but statistically significantly, decreased relative to KK-*A* ^*y*^, possibly reflecting the reduced renal mass in the uninephrectomized mice. 

 While urine markers of renal injury were similar in both KK-*A* ^*y*^ cohorts, renal function appeared to be more significantly impaired in the Unx group. Serum BUN is a routine laboratory test for diagnosis and routine followup of patients with CKD [50] and was elevated in KK-*A* ^*y*^ + Unx mice. Furthermore, glomerular filtration rate (GFR) was decreased by 33% versus nondiabetic KK-*a/a*. Such loss of renal function, roughly comparable to late stage CKD stage 2 [51], suggests that KK-*A* ^*y*^ + Unx mice exhibit the progressive renal injury characteristic of human diabetic nephropathy. 

Consistent with previous reports [20, 21], nonnephrectomized KK-*A* ^*y*^ mice displayed significant glomerulopathy and mild to moderate tubulointerstitial pathology. KK-*A* ^*y*^ + Unx animals had higher median glomerular pathology scores, indicating that addition of Unx exacerbates the severity of glomerulopathy in this model. While tubulointerstitial pathology is not quantifiably different in the two KK-*A* ^*y*^ groups, expression of the fibrotic markers *Col3a1* and *Mmp9* is statistically significantly elevated, and we note a trend towards increased expression of a number of additional fibrotic genes. 

Both KK-*A* ^*y*^ and KK-*A* ^*y*^ + Unx animals exhibited multifocal podocyte foot process effacement and areas of irregular GBM thickening, but we observed a 28% decrease in the density of WT-1^+^ cells only in the KK-*A* ^*y*^ + Unx group, indicating podocyte loss in these mice. Reduced RNA expression of WT-1 and of the podocyte-specific markers nephrin (nphs1) and podocin (nphs2) in KK-*A* ^*y*^ + Unx further support this conclusion. We note, however, that despite the evidence of podocyte loss in KK-*A* ^*y*^ + Unx mice, the concentration of urine nephrin was similar in both KK-*A* ^*y*^ cohorts, suggesting that, while elevated urine nephrin indicated ongoing glomerular injury, the absolute level of nephrinuria may not distinguish relative degrees of podocyte injury. Alternatively, because reduction of renal mass in KK-*A* ^*y*^ + Unx animals halves the total number of podocytes, potentially contributing to urinary nephrin, equivalent levels of nephrinuria may actually reflect a much higher rate of podocyte loss in KK-*A* ^*y*^ + Unx mice. Nevertheless, taken together, these data attest measurable podocyte loss only in the KK-*A* ^*y*^ + Unx group, suggesting that uninephrectomy is necessary to induce podocyte loss in the KK-*A* ^*y*^ model. 

Our data also indicate that uninephrectomy heightened renal inflammation in this model. Immunostaining for the macrophage marker CD68 revealed a statistically significant increase in CD68^+^macrophages in the interstitium of diabetic mice, with a trend for greater infiltration in the uninephric versus the binephric KK-*A* ^*y*^ group. Furthermore, RNA expression of CD68, as well as of the inflammation-associated markers IL-6 and MCP-1, was significantly greater in the KK-*A* ^*y*^ + Unx group. These data all point to increased renal inflammation in Unx-KK-*A* ^*y*^ mice as a contributory factor in the enhanced renal phenotype in this model. 

The results of this study support a strategy for DN model development, complementary to the AMDCC's recommendation, in which multiple “hits” were layered together to exacerbate renal pathology in mouse models genetically predisposed to diabetes. Such an approach may more realistically capture the interaction between genetics and environment contributing to the complex pathogenesis of diabetic nephropathy.
